# Single-Die-Level MEMS Post-Processing for Prototyping CMOS-Based Neural Probes Combined with Optical Fibers for Optogenetic Neuromodulation

**DOI:** 10.3390/mi17020159

**Published:** 2026-01-26

**Authors:** Gabor Orban, Alberto Perna, Matteo Vincenzi, Raffaele Adamo, Gian Nicola Angotzi, Luca Berdondini, João Filipe Ribeiro

**Affiliations:** 1 Microtechnology for Neuroelectronics Laboratory, Fondazione Istituto Italiano di Tecnologia, Via Morego 30, 16163 Genova, Italy; alberto.perna@iit.it (A.P.); matteo.vincenzi@iit.it (M.V.); raffaele.adamo@iit.it (R.A.); giannicola.angotzi@iit.it (G.N.A.); luca.berdondini@iit.it (L.B.); joao.ribeiro@iit.it (J.F.R.); 2 The Open University Affiliated Research Centre at Istituto Italiano di Tecnologia (ARC@IIT), Via Morego 30, 16163 Genova, Italy

**Keywords:** artifact-free, biosensors prototyping, complementary metal–oxide–semiconductor and micro-electromechanical systems technologies (CMOS–MEMS technology), high-density electrophysiology, multimodal recordings, photoelectric shield, SiNAPS-base neural probe

## Abstract

The integration of complementary metal–oxide–semiconductor (CMOS) and micro-electromechanical systems (MEMSs) technologies for miniaturized biosensor fabrication enables unprecedented spatiotemporal resolution in monitoring the bioelectrical activity of the nervous system. Wafer-level CMOS technology incurs high costs, but multi-project wafer (MPW) runs mitigate this by allowing multiple users to share a single wafer. Still, monolithic CMOS biosensors require specialized surface materials or device geometries incompatible with standard CMOS processes. Performing MEMS post-processing on the few square millimeters available in MPW dies remains a significant challenge. In this paper, we present a MEMS post-processing workflow tailored for CMOS dies that supports both surface material modification and layout shaping for intracortical biosensing applications. To address lithographic limitations on small substrates, we optimized spray-coating photolithography methods that suppress edge effects and enable reliable patterning and lift-off of diverse materials. We fabricated a needle-like, 512-channel simultaneous neural recording active pixel sensor (SiNAPS) technology based neural probe designed for integration with optical fibers for optogenetic studies. To mitigate photoelectric effects induced by light stimulation, we incorporated a photoelectric shield through simple modifications to the photolithography mask. Optical bench testing demonstrated >96% light-shielding effectiveness at 3 mW of light power applied directly to the probe electrodes. In vivo experiments confirmed the probe’s capability for high-resolution electrophysiological measurements.

## 1. Introduction

The evolution of complementary metal–oxide–semiconductor (CMOS) technology has been a cornerstone in the advancement of modern electronics. Known for its high integration density, CMOS has driven the miniaturization and performance enhancement of integrated circuits (ICs) over the past few decades [1,2]. CMOS fabrication on large 8″–12″ silicon wafers enables low-cost, high-volume production with consistent quality, but poses challenges for research prototyping [3,4]. To address this, multi-project wafer (MPW) runs partition the wafer surface into millimeter-scale areas shared among multiple users, providing a cost-effective solution for prototyping and small-scale production [5,6,7].

Standard micro-electromechanical systems (MEMSs) cleanroom processes are typically performed at the wafer level, and adapting them to millimeter-sized substrates introduces significant challenges including sample handling, carrier mounting, and constraints on process uniformity. CMOS dies further complicate CMOS–MEMS integration due to morphological factors (e.g., topography) and microfabrication limitations, such as maximum allowable temperatures and material compatibility, imposed by the underlying circuitry [8,9,10]. Prior research on standard photolithography and low-stress post-processing for implantable microsensors underscores the need for soft-material-based approaches to minimize chemical and mechanical stress during photolithography and electron-beam deposition [11].

The combination of CMOS and MEMSs technologies has opened new possibilities, particularly in biosensing [12]. Biosensors have transformed diagnostic applications ranging from blood analysis and infectious disease detection to environmental monitoring and neural interfacing [13,14]. CMOS–MEMS integration has enabled the development of miniaturized and highly efficient sensing platforms with high-channel count devices (hundreds to thousands of recording sites) to directly interface with neural tissue, representing a major leap forward for the field [15,16,17,18,19]. Monitoring neural activity with such minimally invasive neural probes is essential for advancing our understanding of brain function and neurological disorders. Equally important is the ability to manipulate neuronal activity during electrophysiological recordings. Optogenetics enables such activation or inhibition using genetically expressed opsins, which render selected neurons responsive to specific wavelengths of light [20]. Opsins such as Channelrhodopsin-2 (ChR2), Halorhodopsin (NpHR), Archaerhodopsin (Arch), and red-shifted variants like ChrimsonR exhibit distinct activation wavelengths and power density requirements [21,22,23,24]. Among them, ChR2 is one of the most widely used for neural activation, which is stimulated by blue light (≈470 nm) and typically requires 1–10 mW/mm^2^ at the target tissue [21].

Light can be delivered to the brain through three main strategies: micro-light-emitting diodes (µLEDs), waveguides, and optical fibers. Arrays of chronically implantable µLEDs have demonstrated high-throughput optogenetic perturbation [25], and the combination of electrocorticography (ECoG) electrodes with µLEDs has enabled simultaneous recording and stimulation [26]. µLEDs integrated into silicon probes have also been used for concurrent optical stimulation and electrophysiology [27,28,29], though their intrinsic heat generation remains a concern for intracortical implantation [30]. Waveguides integrated within neural probes mitigate heating problems due to the light being generated away from the tissue. Their use in silicon-based implants has been repeatedly demonstrated [31,32,33,34]. Waveguides have also been integrated into CMOS-based systems, such as the Neuropixels Opto probe, which incorporates dual waveguides for red and blue light [34]. However, this required substantial enhancements to the CMOS post-processing pipeline, including photonic layers, stress-compensation structures, and light-shielding strategies. The added complexity also impacted electrode layout, resulting in a lateral pitch of 48 µm.

From a technological standpoint, the simplest method of delivering light into the brain is with optical fibers [35]. Rigid silica fibers are widely used but can cause long-term tissue damage and gliosis. This issue is being addressed through the development of flexible or stretchable polymer fibers [36]. Tapered fibers further reduce invasiveness and allow controlled light emission along the taper by adjusting the input angle, enabling both focal and broad illumination while minimizing tissue damage [37,38]. When combined with electrodes deposited on the fiber surface, tapered fibers reduce artifacts by optimizing the angle between the emitted light and electrode surface [39].

A major challenge in combining optical stimulation with electrophysiological recording (devices called optrodes) is the generation of photoelectric artifacts when stimulating light reaches the metal surface of recording electrodes and routing lines [40,41]. Several strategies have been proposed to mitigate these artifacts. Computational methods such as signal blanking, adaptive noise cancellation, and waveform reconstruction can reduce artifacts but may lead to information loss and increased computational load [42,43,44]. Hardware-based strategies aim to prevent the generation of artifacts altogether. These include geometrically shielding electrodes by orienting light sources so that electrodes remain in shadow [45], or using transparent, non-metal electrodes such as graphene [46,47,48], indium tin oxide (ITO) [49,50], poly(3,4-ethylenedioxythiophene) polystyrene sulfonate (PEDOT:PSS) [51,52], or glass [53,54]. Another widely explored approach is introducing a photoelectric shield layer between the electrodes and the stimulating light. Single-electrode devices have incorporated multilayer internal-reflection structures [55], though these require sophisticated architectures and still demand data post-processing to remove residual artifacts. In passive silicon multielectrode probes, multi-metal shielding layers combined with heavily boron-doped substrates and transient light-pulse shaping have demonstrated significant artifact suppression, though no single strategy proves sufficient alone [29]. Other studies have investigated grounded metal layers to mitigate artifacts generated by waveguide-based or LED-based illumination systems [56,57,58]. Alternatively, modulating the stimulation waveform can reduce artifact generation, as demonstrated through transient pulse shaping or sinusoidal stimulation [29,59].

**Figure 1:**
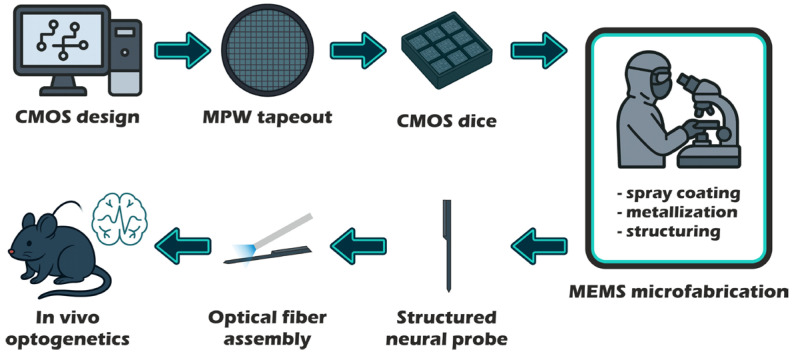
Prototyping production workflow for high-density neural implants made from die-level manufacturing of CMOS ICs (illustrations generated with the help of ChatGPT-4o, a tool developed by OpenAI).

In this paper, we present a CMOS-based neural probe derived from our simultaneous neural recording active pixel sensor (SiNAPS) technology [18,60,61,62], designed for combining with tapered optical fibers to enable concurrent electrophysiology and optogenetic stimulation. To this aim, we used a SiNAPS probe incorporating 512 electrodes with <30 µm pitch, arranged in a four-column configuration within a 150 µm wide and 30 µm thick shank. The narrow probe base (310 µm) facilitated subsequent fiber assembly (see Figure 1 for the prototyping workflow). We describe the detailed die-level MEMS post-processing of the CMOS chip, including the implementation of a photoelectric metal shield requiring only photolithography mask modifications. Optical bench testing demonstrated >96% light-shielding effectiveness at 3 mW of light power applied directly to the probe electrodes, underscoring its potential for artifact-free optogenetic recordings. Finally, we present an in vivo experiment demonstrating the probe’s capability for high-density electrophysiological recordings and its potential for integration into optogenetic research frameworks.

## 2. Materials and Methods

### 2.1. Complementary Metal–Oxide–Semiconductor (CMOS) Design

The SiNAPS probes prototypes were fabricated using the TSMC (Taiwan Semiconductor Manufacturing Company, Hsinchu, Taiwan) 180 nm CMOS technology node and delivered as single dies measuring 3.4 mm × 8.5 mm with a thickness of 152.4 μm as part of an MPW run. Each die contained three distinct neural probe designs to maximize cost-effectiveness during prototyping: a 64-channel ChroMOS probe [17], a 256-channel SiNAPS probe [60], and a 512-channel SiNAPS probe intended for combined electrophysiology and optogenetics, which is the focus of this research. This latter probe incorporates 14 μm × 14 μm electrodes with <30 μm pitch, arranged in a four-column configuration along a 150 μm wide shank and a 310 μm wide base.

The CMOS circuit architecture has been described in detail elsewhere [17,18,60,61]. Briefly, each electrode integrates a dedicated amplifier, and groups of 32 electrodes are locally multiplexed to form a module. This architecture supports rapid prototyping of new probe configurations and enables simultaneous acquisition from all electrodes at 20 kHz each. A second stage multiplexing step further reduces I/O pads requirements, providing one output line per 64 electrodes [61]. Additionally, the SiNAPS technology does not allow access to individual electrodes for electrochemical procedures, but instead incorporates a probe working electrode (PWE) that connects all the electrodes in parallel, bypassing the electrodes’ amplifiers and enabling simultaneous electrochemical procedures for all the electrodes.

During the CMOS design phase, the layout was optimized to accommodate the constraints of subsequent MEMS post-processing, including placement of lithography alignment marks and definition of non-metal regions for dry etching (see Figure S1). The probe geometry was also designed to facilitate later assembly with a tapered optical fiber, enabling concurrent electrophysiological and optogenetic experiments [62].

### 2.2. MEMS Post-Processing of Single CMOS Dies

**Figure 2:**
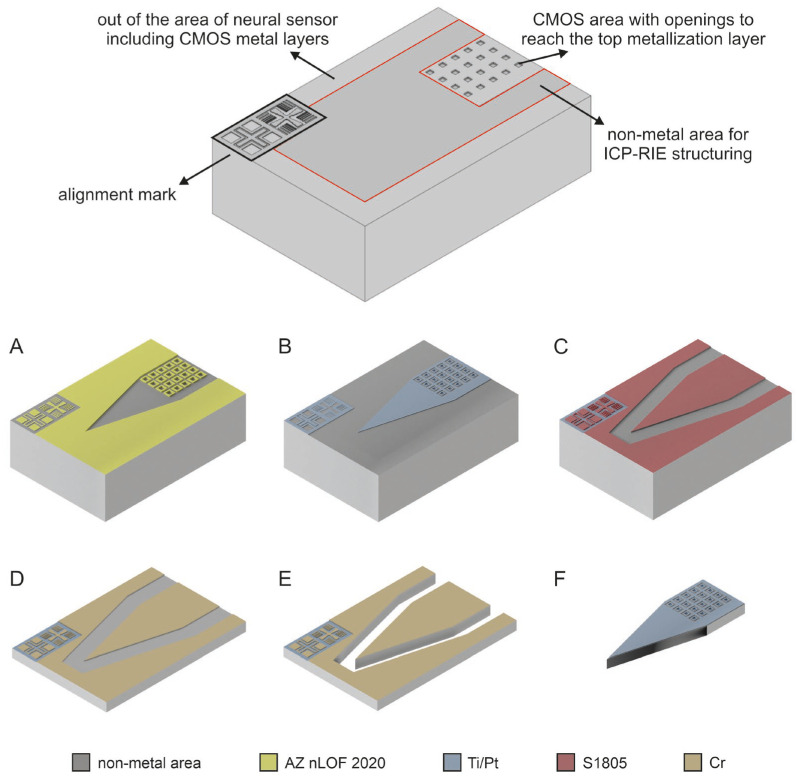
Schematic 3D design of a portion of the CMOS dies near the tip of the 512-channel SiNAPS probe, highlighting the considerations for MEMS microfabrication, such as the placement of the alignment marks and the non-metal areas. The MEMS post-processing was optimized to be performed on CMOS die of 3.4 mm × 8.5 mm, addressing the issue of die-level photoresist coating by the development and optimization of spray-coated photoresist layers, followed by these microfabrication steps: (**A**): Deposition and patterning of the AZ nLOF 2020 negative photoresist for lift-off; (**B**): deposition of the Ti/Pt (10 nm/100 nm) by e-beam evaporation and subsequent lift-off to pattern the electrodes and the photoelectric shield; (**C**): sputter deposition of Cr (400 nm) and patterning of S1805 photoresist; (**D**): Cr wet etching, photoresist removal, and deposition of SiO_2_ (30 nm) for surface protection during the back-side grinding (until reaching 30 µm thick die); (**E**): front-side dry etching to release the structured neural probe; (**F**): Cr wet etching and extensive rinsing in deionized water.

To enable intracortical brain implantation, a series of MEMS post-processing steps were developed and optimized to convert the MPW CMOS dies into fully functional neural probes. One critical aspect of the die-level MEMS post-processing is the uniform and reproducible photoresist coating of single dies for photolithography steps. To address this challenge, we applied an optimized spray-coating technique for specific photoresists. Based on this development, the main microfabrication sequence consisted of two photolithography steps (described in Section Optimization of Spray-Coating for Photoresists Deposition), two metal depositions, backside die grinding, and a final dry-etching step, as summarized in the microfabrication flow of Figure 2.

The first operation involved defining the metal electrodes and photoelectric shield. The CMOS dies included 10 µm × 10 µm openings in the top passivation layer to expose the underlying recording sites. However, the native top metal of the CMOS process is an aluminum–copper (Al/Cu) alloy, which is unsuitable for direct neural interfacing. Therefore, a biocompatible electrode metallization was required [63,64]. A lift-off process using AZ nLOF 2020 negative photoresist (Merck Performance Materials, Darmstadt, Germany) was employed to deposit a 10 nm/100 nm titanium/platinum (Ti/Pt) stack by thermal e-beam deposition (Figure 2A,B). The photoelectric shield, required for combined electrophysiology and optogenetics experiments [62], was patterned in the same step. Electrodes were designed to be 14 µm × 14 µm to ensure complete Al/Cu coverage, with a horizontal pitch of 28 µm, vertical pitch of 29 µm, and a minimum spacing of 4 µm from the photoelectric shield to ensure electrical insulation.

After defining the electrodes and the photoelectric shield, the overall probe geometry was patterned during the second photolithography step (Figure 2C,D). A 400 nm chromium (Cr) layer was deposited by DC sputtering and patterned using S1805 photoresist (Kayaku Advanced Materials, Westborough, MA, USA) and Cr wet etching. Prior to backside grinding, a 30 nm SiO_2_ protective layer was deposited by low-temperature (80 °C) plasma-assisted atomic layer deposition (PA-ALD) to ensure surface uniformity and integrity of the CMOS circuitry. The Cr layer exhibited strong adhesion to the grinding UV tape, and the added SiO_2_ layer facilitated die release after thinning. Additionally, it prevented contamination from the tape adhesive. Since the SiO_2_ layer was only 30 nm thick and the CMOS top passivation is a SiO_2_/SiN_X_ layer, no modifications were required during the subsequent inductively coupled plasma reactive ion etching (ICP-RIE) steps (Figure 2E).

Following die thinning to 30 µm (± 1 µm) and release from the UV tape in acetone, each sample was mounted using Crystalbond (Crystalbond 555 HMP, Aremco Products, Inc., Valley Cottage, NY, USA) on an aluminum ICP-RIE holder. On the non-metal area, which was not protected by the patterned Cr layer (i.e., the probe outlines), the CMOS passivation was etched at 0 °C using a CHF_3_/SF_6_-based ICP-RIE recipe, achieving an etch depth of approximately 12 µm. A standard Bosch process at 5 °C was then used to etch the remaining 18 µm. After completion of the dry etching, the samples were released from the chuck in 90 °C distilled water, allowing the individual dies to separate cleanly, an important consideration given their extreme fragility. Finally, the Cr layer was removed by wet etching, and the samples were thoroughly rinsed in deionized water (Figure 2F). The completed neural probes were then ready for assembly onto custom-designed printed circuit boards (PCBs) for electrical characterization.

#### Optimization of Spray-Coating for Photoresists Deposition

A key challenge in the MEMS post-processing of CMOS dies is the uniform deposition of photoresists. The small size of the dies, only a few millimeters square, and the need to use the entire die surface for circuitry make traditional spin coating unreliable due to the well-known edge effect. This phenomenon results in a thicker resist layer near the substrate edges caused by centrifugal forces during spin coating [11,65,66]. To overcome this limitation, we developed and optimized a spray-coating process suitable for prototyping applications to deposit both AZ nLOF 2020 negative photoresist (for lift-off processes) and S1805 positive photoresist, thereby enabling compatibility with standard photoresist-based microfabrication workflows. For this purpose, we employed an ultrasonic spray coater (ND-SP Spray Coater, Nadetech Innovations S.L., Noain, Spain), originally designed for polymer and nanoparticle coatings.

**Figure 3:**
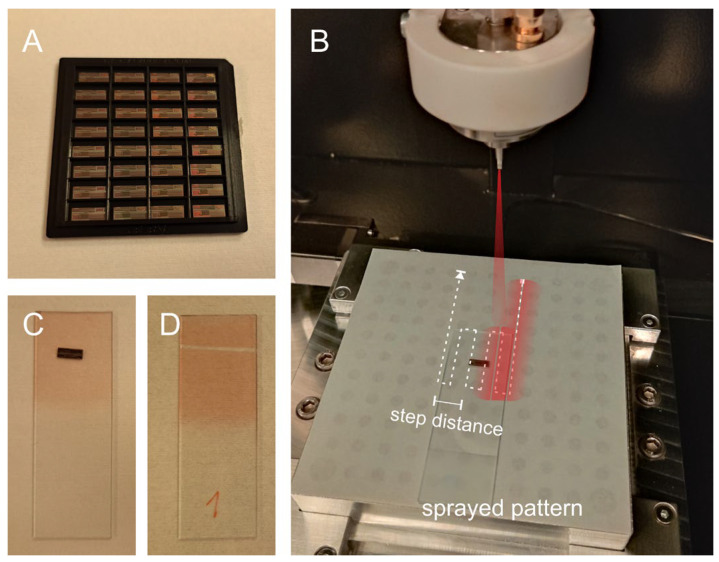
(**A**): CMOS dies 3.4 mm × 8.5 mm as received from the foundry. (**B**): Photoresist spray-coating pattern on a CMOS die mounted on a glass slide. (**C**): S1805 photoresist deposited on the die surface. (**D**): Glass-side sprayed with the same pattern for measuring the layer thickness.

The MEMS post-processing workflow began with the CMOS dies received from the foundry (Figure 3A). In preparation for the first photolithography step, each die was mounted onto a microscope glass slide using water-soluble Crystalbond (Figure 3B), which provided a convenient handling platform throughout the lithographic procedures. Before processing the actual samples, the spray-coating parameters required optimization. The first variable addressed was the viscosity of the photoresists since due to the nozzle diameter of the spray system being fixed, the resists had to be diluted to ensure proper spray without clogging, while avoiding excessive dilution that would compromise film density.

After establishing suitable resist viscosities, the operational parameters of the ultrasonic spray coater (nozzle frequency, power, and air pressure) and the syringe pump settings (flow rate, spraying speed, number of passes, step spacing, and nozzle-to-substrate distance) were optimized. Here, “distance between steps”, refers to the lateral distance between successive sprayed lines on the substrate (Figure 3B), while the nozzle height was defined relative to a 0 mm setting corresponding to its maximum separation from the substrate (70 mm). Achieving a thin yet uniform layer was essential for reliable adhesion to the CMOS dies, as shown in the example of Figure 3C.

To evaluate coating uniformity, preliminary tests were conducted on sprayed blank glass slides (Figure 3D), and the resulting film thicknesses were measured using a stylus surface profiler (Dektak 150, Veeco Instruments Inc., Plainview, TX, USA). These characterization steps guided the final parameter settings used for consistent and reproducible photoresist deposition on the CMOS die samples.

### 2.3. Probe Assembly and Electrodes Electrochemistry

The 512-channel SiNAPS probes were assembled onto custom-designed PCBs for electrical interfacing with a dedicated data acquisition system that was used for both benchtop testing and in vivo acute recordings. Each PCB included wire-bonding pads positioned adjacent to the probe mounting area, passive components (resistors and capacitors) for low-pass filtering, and a Samtec connector (Samtec, New Albany, IN, USA) for interfacing with the acquisition system. The acquisition system, based on a field-programmable gate array (FPGA), was developed in the laboratory and is described in detail elsewhere [17,60].

To secure the probe during wire bonding, a thin layer of Loctite super glue (Henkel AG & Co. KGaA, Düsseldorf, Germany) was applied to the PCB surface. Wire bonding was performed using a semi-automatic wedge–wedge system (5630, F&K Delvotec Semiconductor GmbH, Ottobrunn, Germany) with 25 µm diameter gold wire. After bonding, the wires and the surrounding PCB area were encapsulated with a two-component resin (Ostemer 322 Crystal Clear, Mercene Labs AB, Stockholm, Sweden) to protect them from mechanical damage and the biological environment.

Achieving high-quality electrophysiological recordings requires reducing electrode impedance and, consequently, intrinsic noise [67]. To this end, and to reduce electrode impedance variation, we developed a platinum electrodeposition process [63]. Electroplating was carried out using a PGSTAT204 potentiostat/galvanostat (Autolab Metrohm, Herisau, Switzerland) in a three-electrode electrochemical cell comprising a silver/silver-chloride (Ag/AgCl) reference electrode, a platinum counter electrode, and the potentiostat working electrode connected to the PWE, representing all 512 electrodes in parallel. Prior to deposition, the Ti/Pt electrodes were cleaned by cyclic voltammetry in 0.5 mol/L (1 N) sulfuric acid (H_2_SO_4_) to ensure uniform plating. Platinum was then electrodeposited by replacing the electrolyte with the Platinum AP + 4 g/L Pt solution (Technic, Sassuolo, Italy) and applying a fixed current of 15 nA per electrode for 4 h. Electrochemical impedance spectroscopy (EIS) was performed in 0.9% NaCl (saline), and the impedance of individual electrodes was estimated by multiplying the measured value by 512.

Electrode noise was characterized under two conditions: (1) circuit noise, measured by connecting the PWE to ground to exclude the electrode–electrolyte interface; and (2) saline noise, measured by immersing the probe in grounded saline. In both cases, signals were recorded for 1 min. The acquired data were filtered into three standard frequency bands: full-band [1–5000 Hz], LFP [1–300 Hz], and AP [300–5000 Hz]. Noise levels for each band were calculated as the root mean square (RMS) of the filtered signals.

### 2.4. In Vivo Validation of the 512-Channel SiNAPS Neural Probe

The validation of the needle-like 512-channel SiNAPS probe, structured from a CMOS die, was performed in C57BL/6J mice following a previously established protocol [68]. All procedures complied with the Italian Council on Animal Care guidelines (D.Lgs. 26/2014) and were approved by the Italian Ministry of Health.

Anesthesia was induced using an isoflurane–oxygen mixture (4% isoflurane for induction and 1.5% for maintenance throughout the acute experiment). The animal’s head was secured in a stereotaxic frame, and a 2 mm × 2 mm cranial window was opened. Probe implantation was carried out using a linear actuator operating at a constant speed of 2 µm/s until the target coordinates in the prefrontal cortex were reached: anterior/posterior +1.30 mm, medial/lateral +0.40 mm, and dorsal/ventral +2.50 mm, all relative to Bregma. A 150 µm diameter silver wire was inserted into the cortex of the same hemisphere to serve as a reference electrode. Following implantation, a 10-min electrophysiological recording session was performed to validate the neural probe.

A combination of custom MATLAB (R2022b) and Python (3.11) scripts was used to analyze the acquired electrophysiological data. First, a MATLAB script was used to identify and exclude unreliable channels by comparing their AP-band noise. As channels are expected to have comparable noise levels due to the same underlying technology, channels with unrealistic noise values were discarded. To do so, we identified two categories of unreliable channels: (i) those with saturated front-end amplifiers, and, (ii) those with defective or not fully implanted electrodes. These two categories are typically characterized by very low and very high noise values, respectively, compared to those observed over the probe. Therefore, we computed the median noise value and used this as a global reference. We then set a threshold on noise values below 0.5 and over 2.5 times the noise reference, respectively.

Next, a custom Python script based on the SpikeInterface library [69] was used to run spike sorting with Kilosort4 [70]. Quality metrics were then computed, and units with a mean firing rate (MFR) below 0.1 spikes/s were discarded. No additional automated or manual unit curation was performed.

### 2.5. Photoelectrical Shield Characterization

To characterize the efficiency of the photoelectric shield, we compared the light-induced responses of 512-channel SiNAPS probes fabricated with and without the shield. A flat-end optical fiber (200 µm diameter, NA 0.39; Thorlabs, Inc., Newton, NJ, USA) was positioned perpendicular to the probe surface at a distance of 1 mm to maximize laser illumination of the electrode area. A Cobolt 06-MLD 473 nm, 300 mW laser (HÜBNER Photonics, Kassel, Germany) served as the light source. Nine predefined DC optical power levels were applied for 1 min at the fiber tip, ranging from 0.05 mW to 3 mW. Although such high power levels are advantageous for benchtop characterization, optical powers approaching 3 mW would rarely reach the probe surface in vivo due to strong absorption and scattering in brain tissue [71]. Additionally, the tapered optical fiber to be used during implantation imposes an angle between the electrode surface and the light emitted [38].

Experimental data were analyzed using a custom MATLAB script. To quantify the shield’s effectiveness, we computed the ratio of full-band RMS signal values during light-on (at each tested power) to those during light-off conditions. Electrodes exhibiting an RMS variation greater than 2.5% at a given light power, in either shielded or unshielded probes, were classified as affected by light stimulation.

### 2.6. SiNAPS Probe and Tapered Fiber Assembly

The wire-bonding pads on the CMOS chip featured a needle-like layout, allowing electrical connections to be made from two sides, perpendicular to the probe axis, or from a single side, depending on the PCB design. These configurations enabled placement of the optical fiber in close proximity to the neural probe prior to encapsulation of the wire bonds with the two-component resin. Tapered optical fibers will be used to minimize tissue displacement and damage during in vivo implantation [38]. The fibers used in this study had a diameter of 200 μm, a numerical aperture of 0.39, and a tapered tip length of 2.5 mm.

In the two-sided wire-bonding configuration, the optical fiber was attached to a 3D-printed spacer positioned above the wire bonds and secured to the PCB, resulting in a fiber–electrode distance of approximately 1 mm. In the one-sided wire-bonding configuration, a tapered fiber with an extended untapered segment between the tip and the connector was used, allowing closer placement relative to the neural probe. The fiber was manually positioned on the PCB plane, parallel to the SiNAPS probe, at a distance of approximately 0.5 mm. The two-component resin was then applied, as described earlier, not only to protect and secure the wire bonds but also to maintain the fixed spacing between the optical fiber and the neural probe.

## 3. Results

### 3.1. 512-Channel SiNAPS CMOS-Based Neural Probe

A key step in the successful MEMS post-processing of the CMOS dies was the optimization of the spray-coating procedure to ensure uniform deposition of the required photoresists. Adjusting the photoresist viscosity was essential to prevent nozzle clogging while maintaining reliable film formation. Empirically, the optimal dilution ratios (photoresist/acetone) were determined to be 1:2 for AZ nLOF 2020 and 1:1 for S1805. After systematically tuning the available spray-coating parameters, the settings summarized in Table 1 were identified as optimal, yielding thin and uniform photoresist layers suitable for the subsequent photolithographic steps.

**Figure 4:**
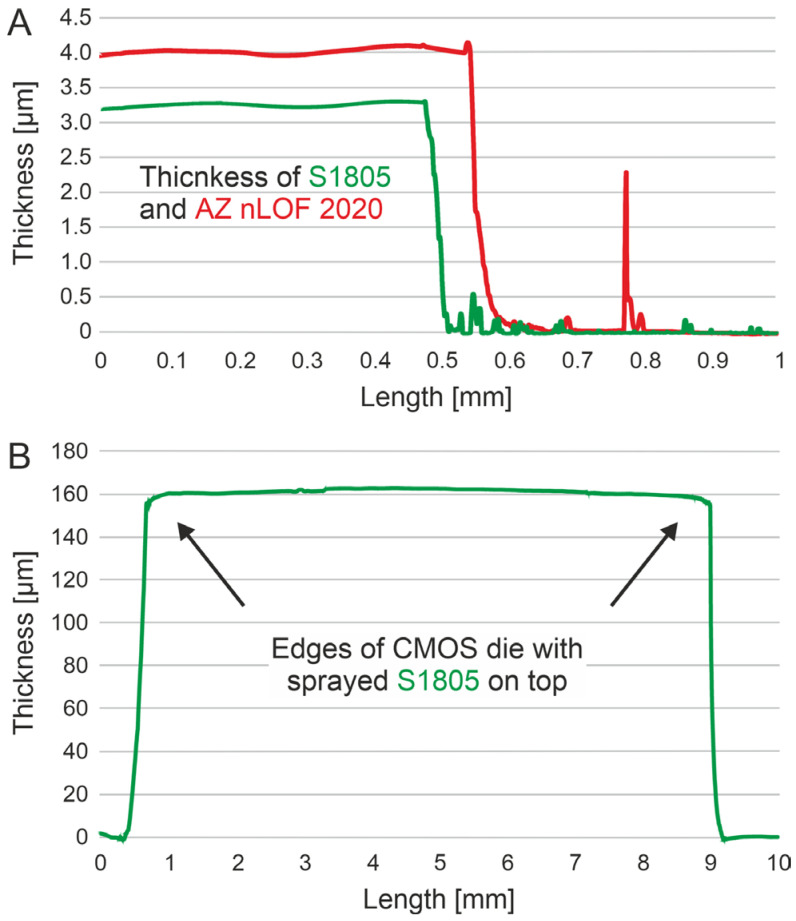
Profilometric measurements: (**A**): Example of thickness measurements for the AZ nLOF 2020 and S1805 photoresists; (**B**): CMOS die with the S1805 photoresist deposited, proving the absence of the edge effect typical from spin-coating depositions. The data shown in red correspond to the AZ nLOF 2020 photoresist and the data in green correspond to the S1805 photoresist.

The photoresist layer thickness was measured by depositing each photoresist onto 10 blank glass slides and sampling two measurement points per slide (*n* = 20 for each photoresist), as illustrated in Figure 4A. The resulting thicknesses were 4.2 µm ± 0.3 µm for AZ nLOF 2020 and 3.2 µm ± 0.3 µm for S1805 photoresists. To further demonstrate the effectiveness of the spray-coating process in mitigating the edge effect, profilometric measurements were performed directly on CMOS dies coated with photoresist (Figure 4B and Figure S2).

**Figure 5:**
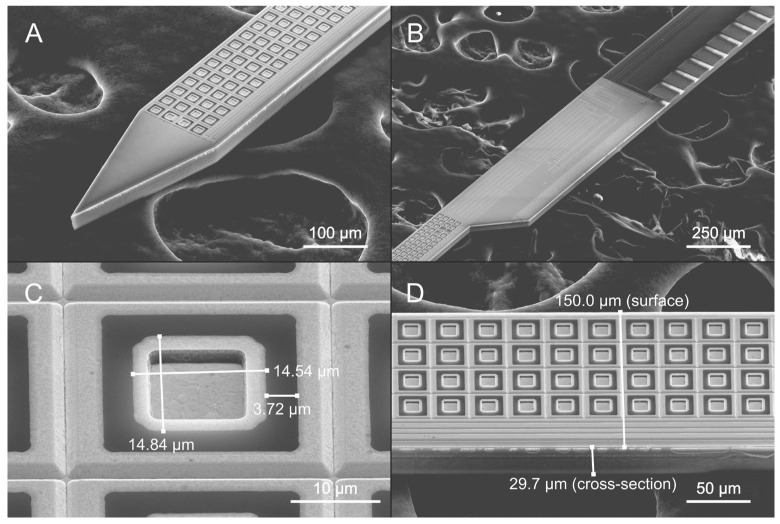
Scanning electron microscope (SEM) images of the realized 512-channel SiNAPS CMOS-based neural probe. (**A**): Probe tip; (**B**): area of the top electrodes and the first wire-bonding pads; (**C**): dimensions of a single electrode and the gap between the electrode and the photoelectric shield; (**D**): probe shank dimensions.

The CMOS–MEMS post-processing enabled by the optimized spray-coated photoresist layers allowed the successful fabrication of the 512-channel SiNAPS neural probes. Owing to the SiNAPS technology [17,18,60,61], which integrates in-pixel amplifiers and in-shank multiplexing, the full set of 512 electrodes can be recorded simultaneously at 20 kHz per channel. Each probe features 14 µm × 14 µm Ti/Pt electrodes arranged in four columns with a horizontal pitch of 28 µm and a vertical pitch of 29 µm, as shown in the scanning electron microscope (SEM) images in Figure 5. The same metal deposition was used to form a photoelectric shield covering the entire probe surface, with a 4 µm separation from the electrode edges to ensure electrical insulation (Figure 5C).

In contrast to other CMOS-based neural probe technologies [34], no modifications to the CMOS circuitry, including electrode pitch, or additional MEMS post-processing steps were required to suppress light-induced artifacts in the electrophysiological signals. Moreover, the simplicity of the approach, which relies only on modifying a photolithography mask, makes it straightforward to adapt any SiNAPS-based neural probe design for use with optical stimulation [72].

The final probe is 30 µm thick, with a 150 µm wide shank and a 310 µm wide base, facilitating assembly with optical fibers. The overall prototyping MEMS post-processing yield from the MPW CMOS dies to the implantable neural devices was approximately 90%, with most failures attributable to mechanical damage during manual handling of the structured devices. Owing to the reliability of standard CMOS technologies, all channels are intrinsically functional, with performance limited only by occasional short circuits between electrodes and the photoelectric shield. These defects originated from glitches during the lift-off process and resulted in approximately 99% functional channels. Further channel functionality is independent from the neural probe fabrication, and on SiNAPS technology, is evaluated directly on the electrophysiological recordings [61].

### 3.2. Benchtop Electrochemical and Electrical Characterization

Following optimization of the Pt electrodeposition process, the electrochemical performance of the electrodes was assessed using EIS. Since the SiNAPS CMOS circuitry does not permit individual electrode impedance measurements, all electrodes were connected in parallel through the PWE, and the measured impedance magnitude was multiplied by the number of electrodes (512). As shown in the Bode plot in Figure S3, the electrodes exhibited an impedance of 425.5 ± 34.2 kΩ at 1 kHz (n = 3 probes × 512 electrodes), confirming their suitability for intracortical electrophysiological recordings [73].

Noise levels were evaluated under two conditions: (1) circuit-only noise, measured by grounding the PWE to exclude electrode–electrolyte interface contributions, and (2) total noise, measured in grounded saline to include electrode interface effects (n = 3 probes × 512 electrodes). Circuit-only noise levels were 16.05 ± 2.76 μV_RMS_ in the full-band [1–5000 Hz], 13.21 ± 3.04 μV_RMS_ in the LFP-band [1–300 Hz], and 8.52 ± 0.39 μV_RMS_ in the AP-band [300–5000 Hz]. Total noise, including electrode–electrolyte contributions, increased to 21.11 ± 0.91 μV_RMS_ (full-band), 17.32 ± 0.88 μV_RMS_ (LFP-band), and 10.69 ± 0.42 μV_RMS_ (AP-band). Overall, the presence of the electrode–electrolyte interface increased the noise by approximately 1.3× relative to the circuit-only condition.

### 3.3. Electrophysiological Recording for In Vivo Validation

**Figure 6:**
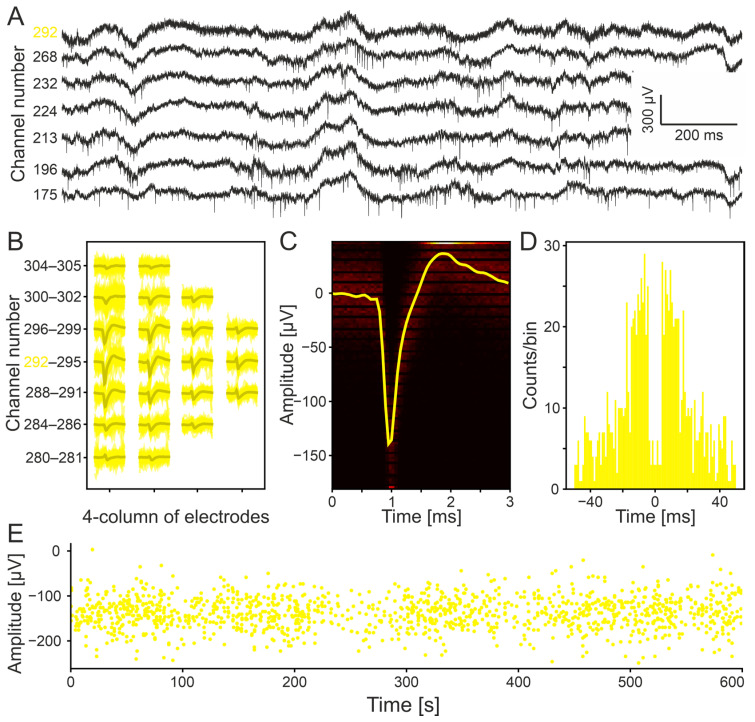
Electrophysiological data recorded by the 512-channel SiNAPS probe developed. (**A**): Full-band data example. (**B**): The 4-column electrode arrangement allows one to track the distribution in space of a sorted single unit. (**C**): The averaged waveform of the same unit. (**D**): Unit autocorrelogram. (**E**): Time stamps versus amplitude of the unit detected during the 10-min-long recording.

An acute in vivo experiment was performed in mice to validate the functionality of the developed 512-channel SiNAPS neural probe. A key objective was to confirm that the photoelectric shield did not impair the recording of neural signals. Figure 6A shows an example of the 10-min full-band electrophysiological recording. After excluding units with an MFR < 0.1 spikes/s and without applying any additional manual or automated curation, Kilosort4 identified 112 single units.

A representative unit detected on channel 292, along with its propagation in neighboring channels, is presented in Figure 6B. Notably, the quad-column layout allows one to clearly observe the spatial distribution of neuronal activity across adjacent electrodes while providing strong potential for identifying the spatiotemporal propagation of light-evoked action potentials in future optogenetic experiments.

The average waveform of the example unit on channel 292 is shown in Figure 6C and its autocorrelogram in Figure 6D. The spike time stamps and amplitudes for the full 10-min recording are illustrated in Figure 6E. A 2-min raster plot extracted from Kilosort4 is provided in Supplementary Figure S4 for all the single units together with their location on the probe; depth coordinate 0 corresponds to the surface of the brain.

### 3.4. Photoelectric Shield Effectiveness and Probe Fiber Assembly

**Figure 7:**
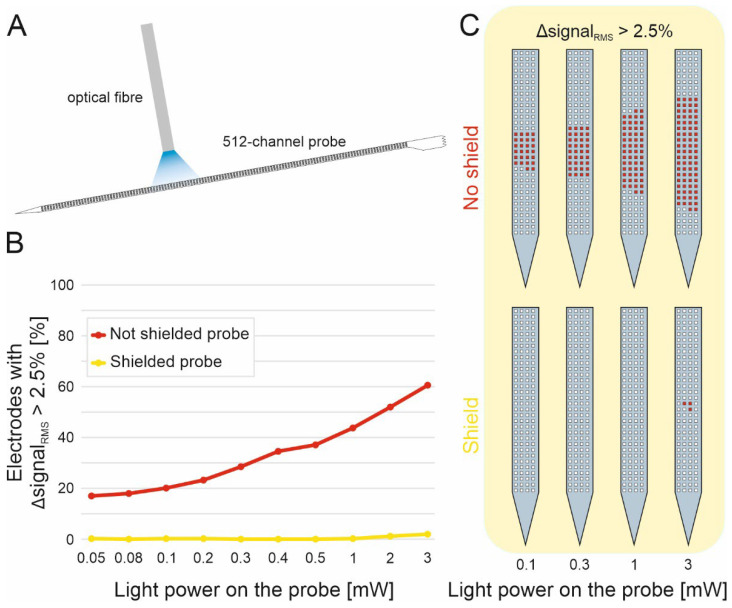
Photoelectric shield effectiveness characterization: (**A**): Illustration of the benchtop setup with a standard optical fiber perpendicularly shining light on the probe electrodes; (**B**): graph comparing the number of electrodes affected by the light stimulation with probes with and without the photoelectric shield (Δsignal_RMS_ > 2.5%) for the full range of optical power tested; (**C**): color map representation of the probe light-affected electrodes (the number of red electrodes are proportional to the Δsignal_RMS_ > 2.5%).

As demonstrated in a previous study [62] and further expanded here, the effectiveness of the photoelectric shield was evaluated using a benchtop setup. A standard 200 µm diameter optical fiber was positioned perpendicular to the probe surface to illuminate the electrodes, representing a worst-case scenario for inducing photoelectric artifacts (Figure 7A). Data were acquired with and without light stimulation for both shielded and unshielded probes, using output light powers ranging from 0.05 mW to 3 mW.

For each electrode, the RMS signal level was computed, and the change between light-off and light-on conditions served as an indicator of charge injection caused by light, i.e., whether the electrode was affected by optical stimulation. A conservative criterion was used, and electrodes exhibiting an RMS increase greater than 2.5% were classified as light affected (Figure 7B). A color map for the principal optical powers is shown in Figure 7C to facilitate interpretation. The complete set of tested light powers and the corresponding percentages of affected electrodes are listed in Supplementary Table S1.

The results show that the photoelectric shield effectively preserves electrode performance, providing >96% light-shielding efficiency at 3 mW of optical power applied directly to the electrode surface. These findings highlight the strong potential of the 512-channel SiNAPS probe combined with the photoelectric shield for achieving artifact-free optogenetic recordings.

**Figure 8:**
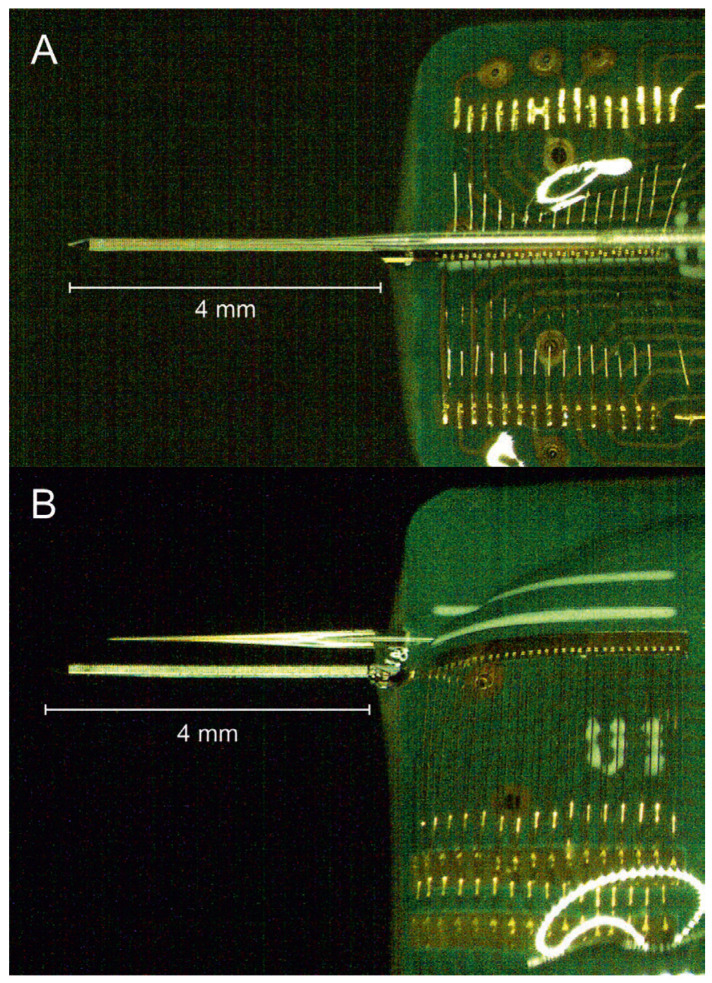
Optical microscopic images of 512-channel SiNAPS neural probe combined with the tapered optical fiber. (**A**): PCB with double-side wire-bonding contacts and the optical fiber assembled on top of the probe. (**B**): PCB with single-side wire-bonding contacts and the optical fiber assembled parallel to the probe (on the PCB surface).

In preparation for future optogenetic experiments (outside the scope of this research) and taking advantage of the narrow probe base (310 µm wide) of the 512-channel SiNAPS probe, Figure 8A,B present two possible strategies for assembling the probe with a tapered optical fiber, creating an optrode. Both configurations allow positioning the fiber tip within 1 mm from the electrode array.

In the first strategy (Figure 8A), the custom PCB is designed for double-sided wire bonding, enabling the optical fiber to be mounted above the probe. In the second strategy (Figure 8B), the PCB incorporates single-sided wire-bonding pads, allowing the optical fiber to be aligned parallel to the probe, on the PCB surface. Both approaches support concurrent high-resolution electrophysiological recording and optical stimulation, enabling future optogenetic experiments. Additionally, we did not observe illumination losses inside the Ostemer 322 Crystal Clear or the shinning of the probe base producing any artifact.

## 4. Conclusions

By developing a reliable and adaptable CMOS–MEMS post-processing workflow at the die level (few-millimeter CMOS substrates), we established a cost-effective prototyping method for miniaturized, high-resolution biosensors. This approach allows us to take advantage of MPW CMOS prototyping processes and to shorten the loop cycle between CMOS circuit design and experimental testing, before scaling up volume production using wafer-level processes. A critical step in this workflow was achieving uniform photoresist deposition, as standard spin-coating techniques suffer from the well-known edge effect when applied to small samples. The spray-coating process developed here enables uniform deposition of both a lift-off compatible negative photoresist (AZ nLOF 2020) and a standard positive photoresist (S1805), allowing compatibility with most cleanroom microfabrication procedures. The approach is limited primarily by the inherent constraints of the CMOS circuitry, such as maximum allowable processing temperatures. The overall post-processing yield was approximately 90%, with most failures attributable to mechanical damage during manual handling.

To demonstrate the robustness of the CMOS–MEMS post-processing pipeline, we developed a 512-channel SiNAPS-based neural probe designed for future assembly with tapered optical fibers to support combined electrophysiology and optogenetics. Leveraging the modularity of the SiNAPS technology developed in the lab, the probe integrates 512 electrodes (14 µm × 14 µm) with <30 µm pitch in a four-column configuration along a 150 µm wide, 30 µm thick shank. A narrow probe base (310 µm) was achieved to facilitate optical fiber integration. Additionally, a metal photoelectric shield was incorporated through simple photolithography mask modifications, without requiring extra material deposition steps. Optical bench testing demonstrated >96% light-shielding effectiveness at 3 mW of optical power applied directly to the electrodes, highlighting the shield’s suitability for artifact-free optogenetic recordings. In vivo experiments further validated the probe’s capability for high-resolution electrophysiological measurements. Finally, we presented assembly strategies for integrating the probe with a tapered optical fiber, establishing a viable route toward complete high-resolution optrodes.

## Figures and Tables

**Table 1:** Optimal process parameters for spray-coating the AZ nLof 2020 and S1805 photoresists.

Ultrasonic nozzle parameters	
Frequency	113,100 Hz (AZ nLOF 2020) 112,900 Hz (S1805)
Power	3.80 W
Air pressure	0.08 Bar
Syringe pump/spraying parameters	
Flow rate	15.00 mL/h
Speed	500.0 mm/min
Number of layers	1
Distance between steps	6.0 mm
Distance from samples	0.0 mm

## Data Availability

Dataset available on request from the authors.

## Supplementary Materials

The following supporting information can be downloaded at: https://www.mdpi.com/article/10.3390/mi17020159/s1, Figure S1: CMOS design of the die used for MEMS microfabrication indicating the design considerations for post-processing: alignment marks placed on die (examples shown in black brackets), non-metal areas (shown in red), and the dimensions of the 512-channel probe used for presenting the process-flow; Figure S2: Profilometric measurement of the CMOS die with the AZ nLOF 2020 photoresist deposited, proving the absence of the edge effect typical from spin-coating depositions; Figure S3: EIS measurement of the 512-channel SiNAPS probe electrodes (n = 3, 3 probes × 512 electrodes). The impedance was estimated by multiplying the module result by 512 and presented 425.5 ± 34.2 kΩ at 1 kHz; Figure S4: Two-minute-long raster plot computed from the detected units by Kilosort 4. The location of the sorted units on the 512-channel SiNAPS probe is shown on the right; Table S1: Percentage of light-affected electrodes of a shielded and not shielded 512-channel SiNAPS probe at the acceptance level of 2.5% compared to the baseline signal RMS at all the observed light powers.

## Abbreviations

The following abbreviations are used in this manuscript:

CMOS	Complementary metal–oxide–semiconductor
MEMSs	Micro-electromechanical systems
MPW	Multi-project wafer
SiNAPS	Simultaneous neural recording active pixel sensor
IC	Integrated circuit
ChR2	Channelrhodopsin-2
NpHR	Halorhodopsin
Arch	Archaerhodopsin
µLED	Micro-light-emitting diode
ECoG	Electrocorticography
ITO	Indium tin oxide
PEDOT:PSS	Poly(3,4-ethylenedioxythiophene) polystyrene sulfonate
ICP-RIE	Inductively coupled plasma reactive ion etching
PA-ALD	Plasma-assisted atomic layer deposition
PCB	Printed circuit board
FPGA	Field-programmable gate array
PWE	Probe working electrode
EIS	Electrochemical impedance spectroscopy
RMS	Root mean square
MFR	Mean firing rate
SEM	Scanning electron microscope
