# Supplementary material for: Single-Die-Level MEMS Post-Processing for Prototyping CMOS-Based Neural Probes Combined with Optical Fibers for Optogenetic Neuromodulation
Source: Micromachines (Basel). 2026 Jan 26;17(2):159. doi: 10.3390/mi17020159 (PMC12942394; doi:10.3390/mi17020159)
Supplement: Supplementary file 1 [file micromachines-17-00159-s001.zip › micromachines-4098693-supplementary.pdf]

micromachines

Supplementary materials

Single-Die-level MEMS Post-Processing for Prototyping CMOS-Based Neural Probes Combined with Optical Fibers for Optogenetic Neuromodulation

Gabor Orban *, Alberto Perna, Matteo Vincenzi, Raffaele Adamo, Gian Nicola Angotzi, Luca Berdondini † and João Filipe Ribeiro †

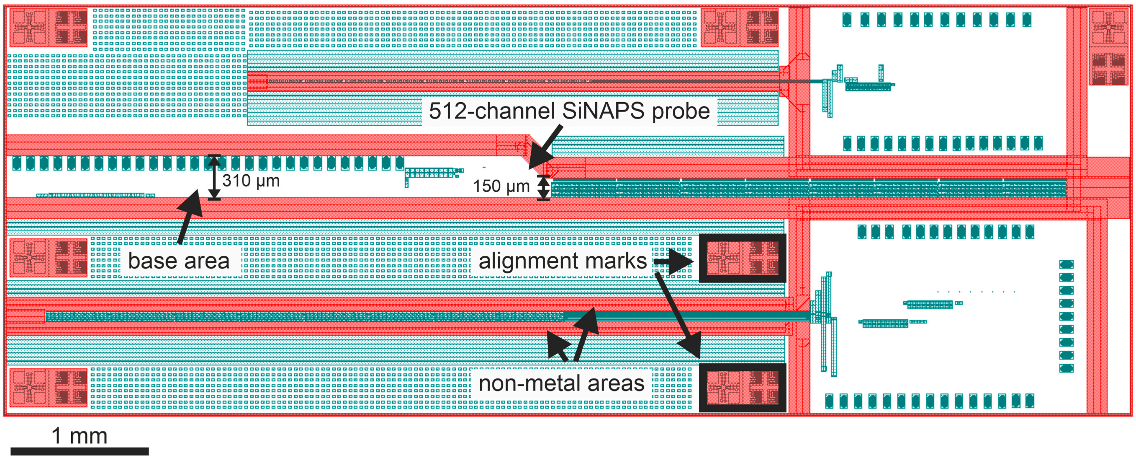

Figure S1. CMOS design of the die used for MEMS microfabrication indicating the design considerations for post-processing: alignment marks placed on die (examples shown in black brackets), non-metal areas (shown in red), and the dimensions of the 512-channel probe used for presenting the process-flow.

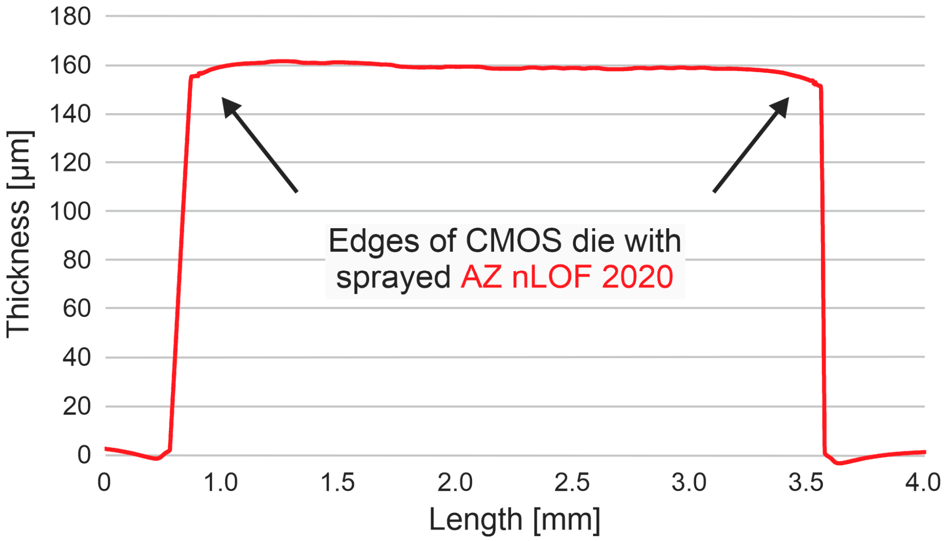

Figure S2. Profilometric measurement of the CMOS die with the AZ nLOF 2020 photoresist, deposited, proving the absence of the edge effect typical from spin-coating depositions.

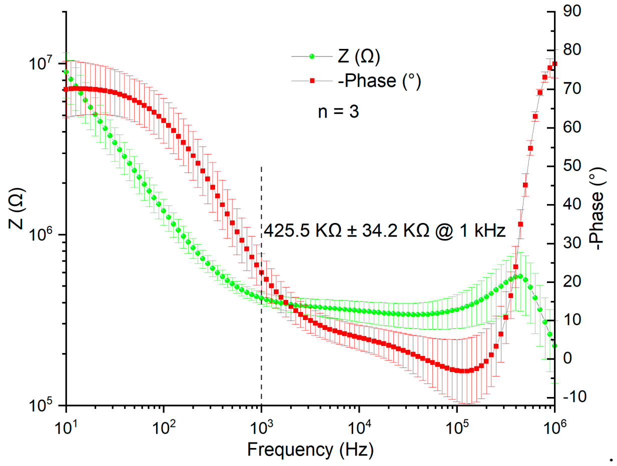

Figure S3. EIS measurement of the 512-channel SiNAPS probe electrodes (n = 3, 3 probes × 512 electrodes). The impedance was estimated by multiplying the mod-ule result by 512 and presented 425.5 ± 34.2 kΩ at 1 kHz.

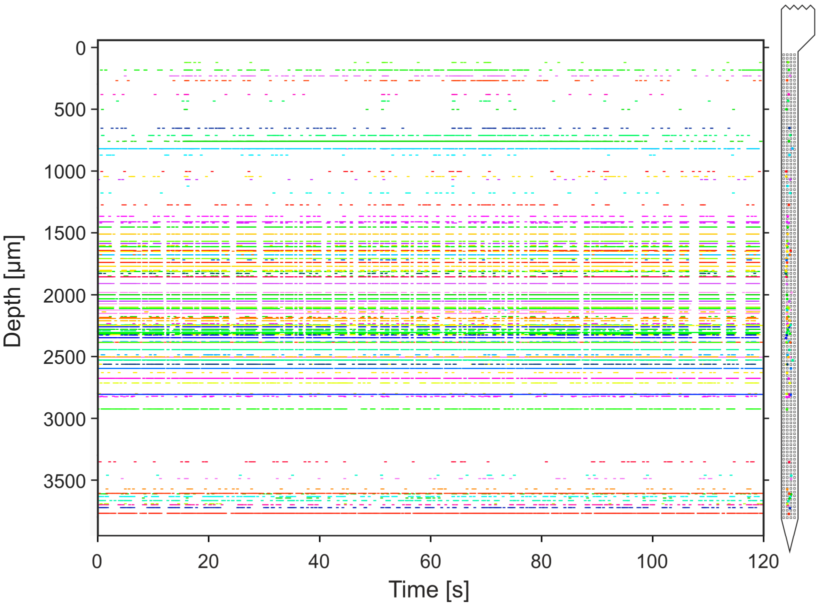

Figure S4. Two-minute-long raster plot computed from the detected units by Kilosort 4. The location of the sorted units on the 512-channel SiNAPS probe is shown on the right.

Table S1. Percentage of light affected electrodes of a shielded and not shielded 512-channels SiNAPS probe at the acceptance level of 2.5% compared to the baseline signal RMS at all the observed light powers.

Percentage of electrodes where $\Delta signal_{RMS} > 2.5\%$												
Not shielded probe	16.99	16.99	17.97	20.12	23.24	25.98	28.52	34.57	37.11	43.75	51.95	60.55
Shielded probe	0.20	0.39	0.00	0.20	0.20	0.00	0.00	0.00	0.00	0.20	1.17	1.95
Light power on the probe [mW]	0.05	0.06	0.08	0.1	0.2	0.25	0.3	0.4	0.5	1	2	3
